# Elevated Levels of *MYB30* in the Phloem Accelerate Flowering in *Arabidopsis* through the Regulation of FLOWERING LOCUS T

**DOI:** 10.1371/journal.pone.0089799

**Published:** 2014-02-25

**Authors:** Liangyu Liu, Jian Zhang, Jessika Adrian, Lionel Gissot, George Coupland, Diqiu Yu, Franziska Turck

**Affiliations:** 1 Key Laboratory of Tropical Forest Ecology, Xishuangbanna Tropical Botanical Garden, Chinese Academy of Sciences, Kunming, Yunnan, China; 2 Max Planck Institute for Plant Breeding Research, Cologne, Germany; 3 State Key Laboratory of Systematic and Evolutionary Botany, Institute of Botany, Chinese Academy of Sciences, Beijing, China; 4 University of Chinese Academy of Sciences, Beijing, China; Universidad Miguel Hernández de Elche, Spain

## Abstract

In *Arabidopsis thaliana*, the R2R3 MYB-like transcription factor MYB30 is a positive regulator of the pathogen-induced hypersensitive response and of brassinosteroid and abscisic acid signaling. Here, we show that *MYB30* expressed under the control of the strong phloem-specific *SUC2* promoter accelerates flowering both in long and short days. Early flowering is mediated by elevated expression of *FLOWERING LOCUS T* (*FT*), which can be observed in the absence and presence of CONSTANS (CO), the main activator of *FT*. CO-independent activation by high MYB30 expression results in *FT* levels that remain below those observed in the wild-type plants, which show an additive CO-dependent activation. In contrast, *TWIN SISTER OF FT* (*TSF*) is repressed in plants expressing high levels of *MYB30* in the phloem. In transient assays, MYB30 and CO additively increase the activity of a reporter construct driven by a 1 kb *FT* promoter. Acceleration of flowering by MYB30 does not require the presence of salicylic acid and is independent of FLC. Taken together, increased levels of *MYB30*, which was reported to be induced in response to the perception of pathogens, can accelerate flowering and *MYB30* may thus be a candidate to mediate cross-talk between gene networks involved in biotic stress perception and flowering time.

## Introduction

Optimal timing of the transition from vegetative to reproductive development is a critical step for the successful reproduction of flowering plants. Independent endogenous and environmental cues combine to determine flowering time through converging pathways. In Arabidopsis, many regulatory inputs are channeled into the transcriptional regulation of *FLOWERING LOCUS T* (*FT*) [1], [2]. *FT* encodes a major component of florigen, which transmits a flowering stimulus from the leaves to the apical meristem [3], [4]. Arabidopsis plants carrying mutations in the *FT* locus flower late in long days (LD), but almost as wild-type plants if grown in short days (SD) [5]. Under inductive conditions, *FT* is transcribed in the companion cells of the distal leaf veins, and FT protein is translocated with the assistance of FT-INTERACTING PROTEIN 1 (FTIP1) to sieve elements [6], [7], [8]. After the long distance movement, FT protein is unloaded to the shoot apical meristem cells, where it is proposed to migrate to the nuclei to form a complex with the bZIP transcription factor FLOWERING LOCUS D (FD) and 14-3-3 proteins [9], [10], [11]. The FT/FD complex reprograms the transcriptional networks in the meristem to induce the floral transition. *SUPPRESSOR OF OVEREXPRESSOR OF CONSTANS* 1 (*SOC1*) and *APETALA1* (*AP1*) are induced in the meristem through FT [3], [10], [11], [12], [13].

Several key activators and repressors of *FT* have been identified. In the photoperiod pathway, CONSTANS (CO) activates *FT* by binding to one of several TGTG(N2-3)AT(G) motifs located in the proximal promoter [7], [14]. CO protein is stabilized at the end of LDs but does not accumulate under SDs [15]. CO activation of *FT* depends on the presence of accessory binding partners belonging to two related histone-fold protein families, NF-YB and NF-YC [16], [17]. The MADS-domain transcription factor FLOWERING LOCUS C (FLC), whose levels decrease gradually during vernalization, represses *FT* by binding to a CArG-box in the first intron in leaves [18], [19], [20]. In ambient low temperature, another MADS-domain factor, SHORT VEGETATIVE PHAGE (SVP), represses *FT* by binding to a CArG-box upstream of the *FT* proximal promoter [21]. FLC and SVP can form a complex and it seems that they can collectively but also individually repress *FT* [20]. In high ambient temperatures, the bHLH domain transcription factor PHYTOCHROME-INTERACTING FACTOR 4 (PIF4) activates flowering dependent on sequences found in the 5′UTR of *FT* [22]. TEMPRANILLO1 (TEM1), an AP2-like protein counteracts the CO-mediated activation of *FT* and directly binds to the *FT* 5′ UTR *in vivo* [23]. Interestingly, GIGANTEA (GI) as an activator of *CO* seems to activate *FT* directly in mesophyll cells by binding to *FT* repressors and associating with the *FT* proximal promoter [24], and also other upstream regulators of *CO* such as FKF1 and CDFs have been reported to directly regulate *FT* based on ChIP results [25]. Similar to *FT*, its closest relative *TWIN SISTER OF FT* (*TSF*) plays a role as flowering integrator [26], [27], [28]. In seedlings, *TSF* is mainly expressed in the vascular tissue of leaf petioles and the hypocotyls [28]. Although *tsf* single mutants do not show obvious later flowering in LD or SD, a loss of *tsf* enhances the *ft* flowering phenotype in LDs and SDs and *TSF* transcription is also responsive to CO [26], [27], [28].

Plant hormones also provide an input to flowering time regulation. Under non-inductive SDs, Gibberellin (GA) plays an important role as demonstrated by the observation that the *ga1* mutant, which is strongly impaired in GA biosynthesis, never flowers [29]. In LDs, overexpression of *GA2OX7*, which encodes a protein that decreases the amount of active GA in vascular leaf tissues delays flowering through decreased *FT* and *TSF* expression [30]. Brassinosteroids (BR) and salicylic acid (SA) are reported to promote flowering at least partially by repressing *FLC* [31]. Plants constitutively expressing the bacterial salicylic acid hydrolase encoding *NahG* gene and the SA-biosynthetic mutant *salicylic acid induction deficient 2* (*sid2*) have been shown to flower late in LDs and SDs [32]. SIZ1, a PIAS-type SUMO E3 ligase related to yeast Siz (SAP and Miz) proteins [33], facilitates SUMO modification of FLD, which represses *FLC* through the autonomous pathway. Plants mutated in *siz1* flower early and express reduced levels of *FLC* indicating that sumoylation is important for FLD’s repressive function [34]. In addition, the early flowering in *siz1* mutant plants is dependent on the presence of SA [34].

Plants are likely also to coordinate the transition to reproductive development with adverse environmental factors such as biotic and abiotic stresses although they may have to differentiate their response to the severity of the stress [35]. However, relatively little is known about the cross-talk and potential overlap between the gene networks regulating flowering time, pathogen defense and response to biotic stress. In a systematic screen for transcription factors that accelerate flowering if highly expressed in the phloem, we identified the transcription factor MYB30 as novel factor with a potential to regulate flowering from the phloem. MYB30 was first characterized for its role in cell death during the hypersensitive response (HR) to pathogens [36]. This R2R3 MYB-like factor is a positive regulator of HR and induces cell death through SA accumulation and the regulation of SA-responsive genes [37], [38], [39]. *MYB30* expression is transiently upregulated during bacterial pathogen recognition [37]. In the BR signaling pathway, MYB30 interacts with BRI1-EMS-SUPPRESSOR 1 (BES1) and, as a cofactor, positively regulates BR-responsive target genes [40]. During germination, MYB30 is a target of SIZ1-mediated sumoylation, which appears to stabilize the protein possibly by protecting it from ubiquitin-mediated degradation [41], [42]. Seeds carrying *myb30* loss-of-function alleles are hypersensitive to ABA indicating that MYB30 acts on the crossroad of yet another hormonal pathway [41]. Taken together, MYB30 is an interesting candidate, which has the potential to mediate cross-talk between the flowering and stress pathways. In the following, we show how MYB30 could integrate into the flowering time regulatory network.

## Materials and Methods

### Plant Growth

For expression studies, seeds were sterilized in 75% ethanol and 100% ethanol for 5 min each and sowed on GM medium supplemented by 1% sucrose. After stratification at 4°C for 3 days, plants were grown in climate chambers at 22°C in LDs (16 hours light/8 hours dark). Time of transfer to climate chambers represents day 0. During the collection of samples, ZEITGEBER TIME (ZT) 16 was in the light and ZT24 was in the dark before the switch of light condition. For flowering time measurements, seeds were sowed on soil and stratified at 4°C for 3 days. Soil trays were transferred to LDs (16 hours light/8 hours dark) or SDs (8 hours light/16 hours dark) Percival cabinets or the green house as indicated.

### Mutant and Transgenic Plants

The *SUC2_prom_::MYB30* fusion was constructed by recombining the *MYB30* open reading frame into a modified *pGREEN* vector in which the *SUC2* promoter was inserted upstream of the GATEWAY recombination site. The original *MYB30-pENTRY* clone containing the full open reading frame was generated by the REGIA consortium [43]. The *MYB30_prom_::GUS* plants were generated by amplifying 3.8 kb of the upstream MYB30 regions with GATEWAY compatible primers and recombining the product into *GW-GUS-pGREEN* vector previously described [7]. The constructs were introduced into *Agrobacterium tumefaciens* GV3101 that carried the *pSOUP* helper plasmid. Transgenic plants were generated using the floral dip method [44]. Only lines that segregated a single locus T-DNA in the T2 generation were further analyzed. For *SUC2_prom_::MYB30*, two of three lines were early flowering of which one was selected for crosses and further analysis. Plants of *ft-10*, *tsf-1*, *co-sail*, *NahG* and *flc-3* are all Colombia-0 ecotype and have been described (http://www.arabidopsis.org/). The *myb30-1* (SALK_027644C) and *myb30-2* (GK_022F04) mutants were ordered from NASC. Primers for plasmid construction and genotyping are listed in Table S1.

### Bombardment

30 µg gold particles were prepared for every 10 bombardments. First, 70% ethanol was used to wash the gold particles followed by three times sterile water. Finally the gold particles were suspended in 500 µl of 50% sterile glycerol.

In each bombardment, 15 µg DNA in total was used including 5 µg *35S_prom_::RedLUC-pJAN*, 5 µg *1.0 kb FT_prom_::GreenLUC-pGREEN* and 5 µg of either *35S_prom_::CO, 35S_prom_::MYB30* or *35S_prom_::GW* cassette vector. The DNA was mixed with 50 µl prepared gold beads, 50 µl 2.5 M CaCl_2_ and 20 µl 0.1 M spermidine. After two washes with ethanol, the DNA-gold mix was resuspended in 50 µl 100% ethanol which was used for two independent bombardments. 5–10 mm Arabidopsis leaves were transformed by the Biolistic™ Particle Delivery System (BIO-RAD, PDS-1000/HE). After 12–24 hours incubation of the samples in constant light conditions, 1 mM luciferin was sprayed on the leaves and after one minute the emitted light was immediately measured with the help of a cooled CCD-camera adapted with optical filters to detect RedLUC and GreenLUC independently. The ratios of GreenLUC/RedLUC signals were calculated with the help of the excel macro Chroma-LUC™ Calculator version 1.0 (Promega).

### Reverse Transcribed Quantitative-PCR (RT-qPCR) and Semi-quantitative PCR

Total RNA was isolated using the RNeasy Mini kit according to instructions (Qiagen, Cat. no. 74104). 5 µg RNA was used for reverse transcription. After treatment with DNA-*free*™ DNAse I according to manufacturer’s instructions (Ambion, Cat. no. AM1906), cDNA was generated at 42°C for 2 hours using Superscript II reverse transcriptase (Life Technologies, Cat. no. 18080-044). The reaction was terminated by incubation at 75°C for 10 min.

Quantitative PCR (qPCR) was performed using a real-time PCR cycler (Roche LC480). 10 µl PCR reactions included cDNA corresponding to 70 ng RNA template, 10 pmole forward primer, 10 pmole reverse primer and 5 µl 2×SYBR Green qPCR buffer (Biorad, Car. no.18080-044). PCR products varied in length of 200∼300 bp, and the program was composed of step 1∶95°C, 3 min; step 2: [95°C 10 sec; 58°C 10 sec; 72°C, 20 sec] 40 cycles; and step 3: [95°C 3 min; 50°C 1 min; rise 0.5°C every 10 sec until up to 95°C]. Absence of primer dimers or unspecific PCR products was confirmed by melting curve analysis. PCR products were quantified against an internal standard generated by diluting a cDNA sample (5-fold dilution series from 1 to 0.008) prepared from *SUC2::MYB30* expressing Col plants grown in LD. Standard deviations were calculated from three technical replicates. *PP2A* was used for sample normalization.

Semi-quantitative PCR was performed using a thermal PCR cycler (Eppendorf). 10 µl PCR reactions included cDNA corresponding to 70 ng RNA template, 10 pmole forward primer andreverse primer, 1×reaction buffer and 0.5 U LA Taq DNA polymerase (Takara). PCR products *MYB30* and *PP2A* were between 200–300 bp, and the program was composed of step 1∶95°C, 3 min; step 2: [95°C 10 sec; 58°C 10 sec; 72°C, 20 sec] 25 cycles.

### GUS Histochemical Staining

Young seedlings were incubated in 90% Acetone on ice for 30 min, rinsed with 50 mM sodium phosphate buffer and incubated for 24 hours at 37°C in GUS staining solution (0.5 mg/ml X-Gluc, 50 mM sodium phosphate buffer, 0.5 mM potassium ferrocyanide, 0.5 mM potassium ferricyanide, 0.1% Triton X-100). After incubation, samples were washed with 50 mM sodium phosphate buffer for 30 min and 70% ethanol several times until leaves turned white. The GUS staining was visualized and photographed under a stereomicroscope (Leica).

## Results

### Strong Expression of *MYB*30 in Phloem Companion Cells Promotes Flowering under LDs and SDs in Arabidopsis

**Figure 1:**
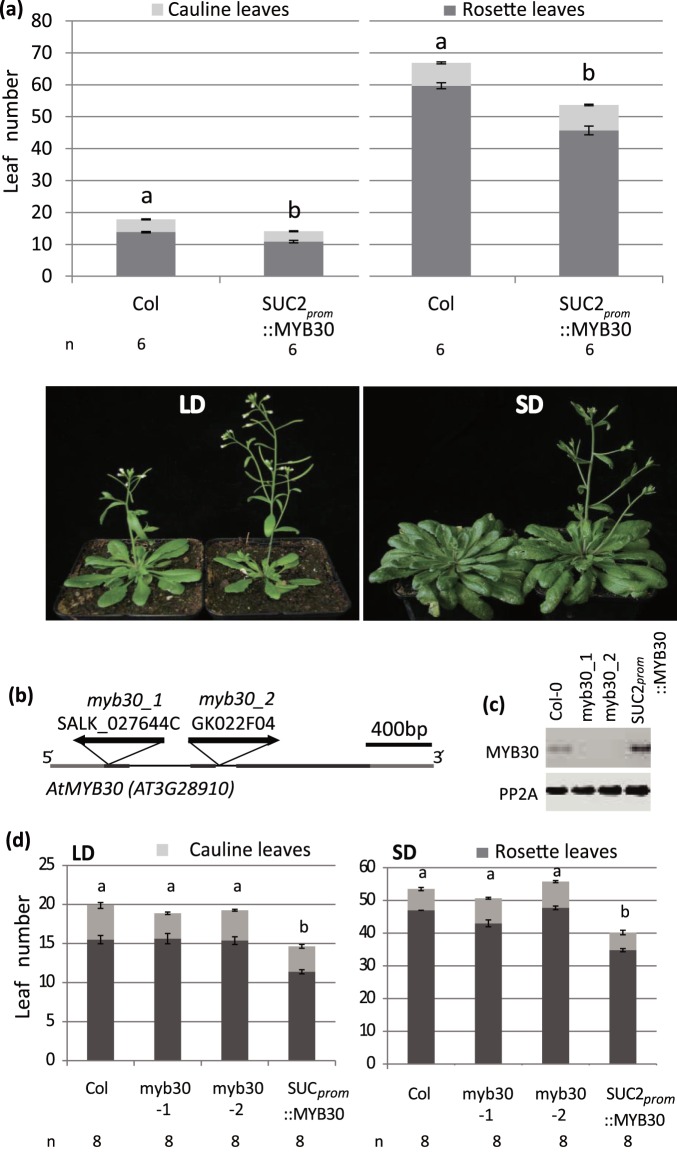
Flowering time of *SUC2_prom_::MYB30* and *myb30* mutant plants in LD and SD conditions. (**a**)WT and *SUC2_prom_::MYB30* expressing Col plants were grown in LDs (16 h light) and SDs (8 h light) in a temperature controlled climate chamber. The number of rosette and cauline leaves was counted to determine flowering time. Pictures were taken after the WT plants had started to bolt in each condition. Statistical significance was determined using the Student’s t-test (p<0.01). Significant differences are indicated by different letters above the bars, SD and LD treatments are tested as separate experiments. (**b**) Position of T-DNA insertion lines of *myb30*. A 400 bp PCR fragment used to detect expression is indicated. (**c**) Semi-quantitative RT-PCR confirms *myb30* mutants as loss-of function alleles. (**d**) Flowering time of Col, *myb30_1*, *myb30_2* and *SUC2_prom_::MYB30* plants were measured in LD (left) and SD (right) conditions in a temperature controlled climate chamber. Statistical significance was determined using the Student’s t-test by comparing each genotype to the respective Col control (p<0.01). Significant differences are indicated by different letters above the bars. The number of plants for each genotype (n) is indicated below the graph.

The key flowering time components of the photoperiod pathway, *CO* and *FT,* are expressed in phloem companion cells of the leaf [6], [7], [45]. In a flowering time screen under LD conditions we used the *SUCROSE-PROTON SYMPORTER 2* (*SUC2*) promoter, which drives expression in all phloem companion cells, to identify novel factors from a library of 800 transcription factors (TF) that have a potential to influence flowering from the phloem [46]; [43], [47], [48]. Two independent *SUC2_prom_::MYB30* transformed lines (#1 and #2) flowered earlier than wild type (WT) in LD growth conditions (Figure S1). We selected line #2 for further analysis and confirmed the earlier flowering phenotype, which was even more pronounced under SDs compared to Col plants grown in the same condition (Figure 1a). To establish that *MYB30* (*AT3G28910*) was expressed in phloem companion cells in wild-type plants, we generated *MYB30_prom_::GUS* reporter lines in the Col background. Strong GUS signal was observed in vascular tissues of leaves, hypocotyl and roots, but expression was not restricted to the phloem (Figure S2). In our growth conditions, two independent T-DNA insertion lines of *myb30* (Figure 1b), which did not express full length *MYB30* transcript (Figure 1c) showed similar flowering time to WT under both LD and SD conditions showing that MYB30 is not required to promote flowering in WT plants under these conditions (Figure 1d).

### Overexpression of *MYB30* in the Phloem Increases *FT* and Reduces *TSF* Levels

**Figure 2:**
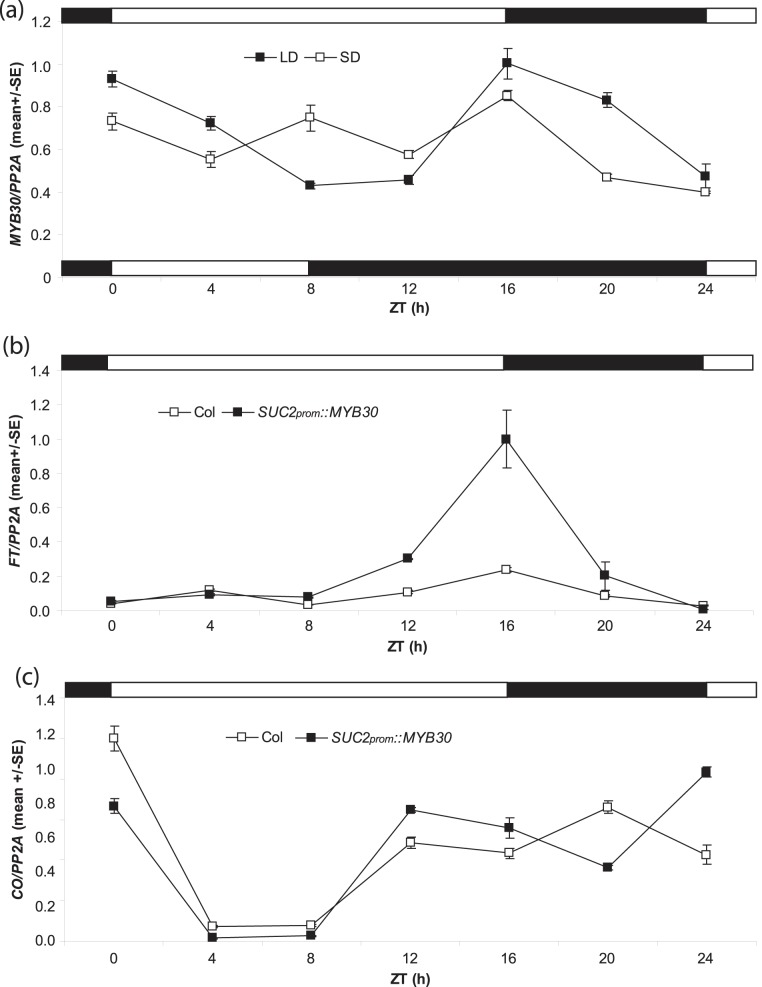
Diurnal expression of *MYB30*, *FT* and *CO*. (**a**) Diurnal expression pattern of *MYB30* was measured in Col grown in LDs and SDs by RT-qPCR. Total RNA was prepared from 10-day old seedlings sampled every 4 hours (ZT0-ZT24). (**b**) *FT* expression was measured comparing WT to *SUC2_prom_::MYB30* expressing Col plants. 10-day-plants in LDs were collected every 4 hours from ZT0-ZT24. (**c**) *CO* expression was measured comparing WT to *SUC2_prom_::MYB30* expressing Col plants. 10-day-plants in LDs were collected every 4 hours from ZT0-ZT24. Error bars represent the standard error of three technical replicates relative to the expression of *PHOSPHATASE 2A* (*PP2A*). The experiment was repeated three times with similar results.

Day-time specific expression is a key feature of the photoperiodic pathway genes *CO* and *FT*. To test whether *MYB30* transcripts accumulate rhythmically, we measured steady-state mRNA levels during 24 h cycles in LDs and SDs. WT seedlings showed a diurnal rhythm of *MYB30* transcripts with a peak at ZT16 and a relatively similar pattern under LDs and SDs (Figure 2a). Since transcript levels were high throughout the day despite the observed diurnal pattern, MYB30 is likely to be present throughout the day unless the protein is subjected to post-transcriptional regulation. In LDs, *MYB30*’s highest expression overlaps with the strongest accumulation of CO protein.

Next, we tested whether expression of flowering time marker genes such as *FT*, *TSF* and *CO* changed in *SUC2_prom_::MYB30* plants. We measured steady-state mRNA levels during 24 h cycles in LDs. *FT* transcripts at ZT16 were ∼5 times enriched comparing *SUC2_prom_::MYB30* to wild-type Col plants (Figure 2b and Figure S3a). An increase of *FT* expression can likely contribute to the earlier flowering time of *SUC2_prom_::MYB30* under LDs. In contrast, *CO* mRNA did not change significantly, especially during daytime (Figure 2c and Figure S3b). This suggested that *SUC2_prom_::MYB30* activated flowering through transcriptional regulation of *FT* that was not conveyed by changes in *CO* mRNA levels.

**Figure 3:**
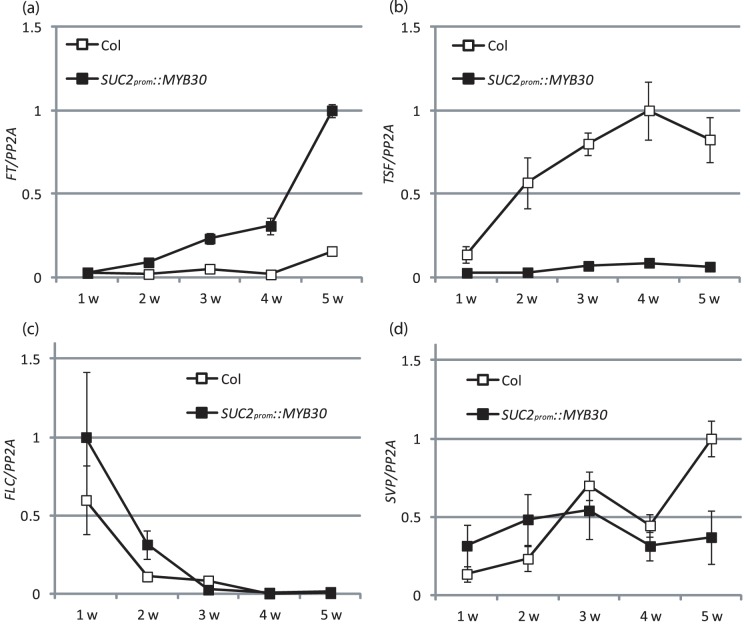
Expression of flowering time genes in *SUC2* *_prom_* *::MYB30* expressing plants during development. Material was collected once per week at ZT16 from WT and *SUC2_prom_::MYB30* expressing Col plants grown in LDs in climate chamber. Samples of 1 week and 2 week old plants were collected from all aerial parts, those of 3–5 weeks from leaves. Expression levels were measured by RT-qPCR for *FT* (**a**), *TSF* (**b**), *FLC* (**c**), and *SVP* (**d**). Values are shown as Mean ± SD after normalization of expression with values obtained for *PP2A* for technical triplicates. A biological replicate of the experiment gave similar results.

We also measured the expression level of different flowering-time genes during the course of Arabidopsis development. With increasing age from one to five weeks, *FT* mRNA transcripts accumulated to significantly higher levels in *SUC2_prom_::MYB30* plants (Figure 3a), whereas *SVP* and *FLC* mRNA did not obviously differentiate the genotypes during the first 4 weeks. Therefore, increase of *FT* mRNA by strong *MYB30* expression in the phloem does not correlate with reduced *FLC* and *SVP* levels. At 5 weeks SVP is differentially expressed in *SUC2_prom_::MYB30* and Col plants, which could be due to earlier transition into flowering that is observed in the transgenic plants. Surprisingly, *TSF* mRNA showed a pattern opposite to *FT* in that it was down-regulated in *SUC2_prom_::MYB30* plants during development (Figure 3b). Thus, *FT* and *TSF*, which are both part of ‘florigen’ perform agonistically in *SUC2_prom_::MYB30* expressing plants grown in LDs.

### Ectopic Expression of *MYB*30 Accelerates Flowering and Impacts *FT* and *TSF* Expression in Absence and Presence of *CO*

**Figure 4:**
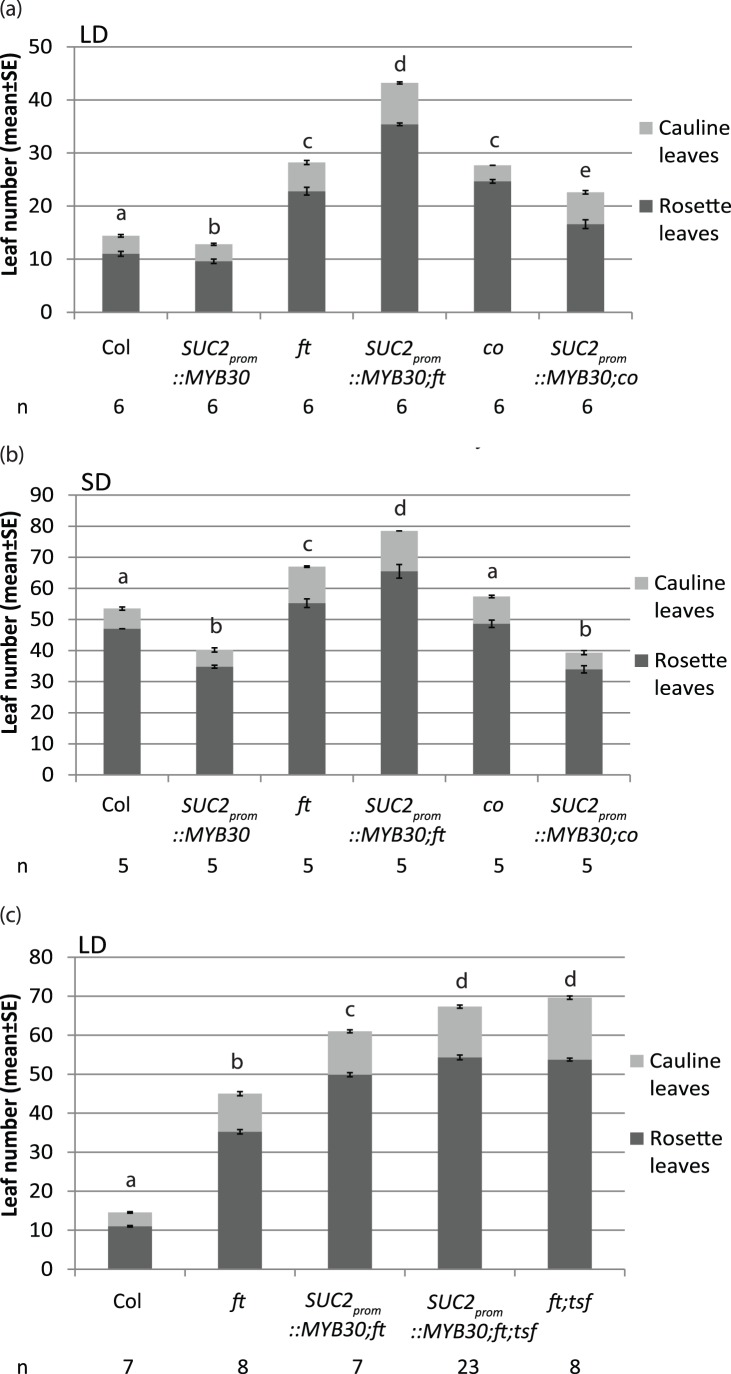
*SUC2_prom_::MYB30* expression accelerates flowering dependent on *FT* and independent on *CO*. Flowering time measurement of Col, *SUC2_prom_::MYB30*, *ft*, *SUC2_prom_::MYB30;ft*, *co* and *SUC2_prom_::MYB30;co* under LDs (**a**) and SDs (**b**) in the greenhouse. Plants *SUC::MYB30*;*ft* and *SUC2_prom_::MYB30*;*ft;tsf* were grown in LDs greenhouse (**c**). Statistical significance was determined using one way Analysis of Variance (ANOVA) followed by multiple comparison of Holm-Sidak method (p<0.01). Significant differences are indicated by different letters above the bars. The number of plants for each genotype (n) is indicated below the graph.

In the photoperiod pathway, *CO* is the major activator of *FT* and *TSF* [26]. To answer whether ectopic expression of *MYB30* accelerates flowering via the photoperiod pathway, we crossed *SUC2_prom_::MYB30* plants to *ft* and *co* mutants. *SUC2_prom_::MYB30;ft* double mutants flowered even later than *ft* single mutants under LDs, whereas *SUC2_prom_::MYB30;co* flowered obviously earlier than *co* mutants (Figure 4a). This corroborated the observation that *MYB30* acts upstream of *FT* and suggested that other genes are affected that caused an enhanced late flowering phenotype in the *ft-10* mutants. Since ectopic expression of *MYB30* can accelerate flowering in the *co* mutant, it can be concluded that MYB30 does not require CO to impact flowering although plants with defective *co* flowered much later than wild-type plants. Accelerated flowering in *SUC2_prom_::MYB30* was also observed under SDs (Figure 4b), which indicated that MYB30 can promote flowering independent of the photoperiod.

*SUC2_prom_::MYB30;ft* flowered later than *ft* plants in both LD and SD growth conditions (Figure 4a, b). Considering that *TSF* was down-regulated in *SUC2_prom_::MYB30*, *TSF* was a candidate gene to explain an enhanced late flowering phenotype in *SUC2_prom_::MYB30;ft* plants. To further test this, we generated *SUC2_prom_::MYB30;ft;tsf* triple mutants, which showed a flowering time that was not significantly different to that of *ft;tsf* double mutants (Figure 4c and Figure S4). This confirms that altered *TSF* transcription likely explains the flowering time differences between *ft*, *SUC2_prom_::MYB30;ft* and *SUC2_prom_::MYB30;ft;tsf* plants. In sum, *FT* and *TSF* are both downstream factors of *MYB30*, but while *FT* is activated, *TSF* is repressed.

**Figure 5:**
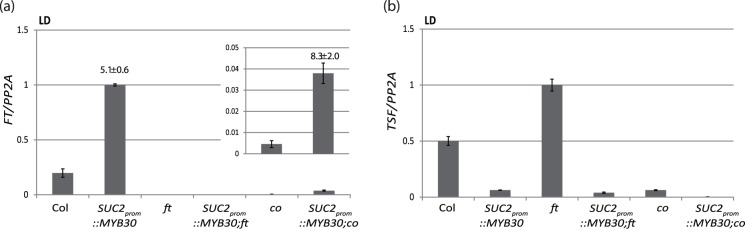
Expression of *FT* and *TSF* in WT, *ft* and *co* plants. Material from 13-day-seedlings grown on soil in LDs in the greenhouse was collected at ZT16. *FT* (**a**) and *TSF* mRNA (**b**) levels were determined by RT-qPCR in different genotypes as indicated. Insert in (**a**) shows values for *co* and *SUC2_prom_::MYB30;co* at a lower scale. Fold-change *FT* expression in the *SUC2_prom_::*MYB30 lines compared to the respective control is indicated above the bar. Values are shown as Mean ± SD after normalization of expression with values obtained for *PP2A*. Experiments were repeated twice with similar results.

*FT* transcript levels were ∼8 times higher in *SUC2_prom_::MYB30;co* plants compared to *co* single mutants under LDs, which is similar to the ∼5-fold increase observed in *SUC2_prom_::MYB30* plants compared to WT (Figure 5a). Therefore, the effect of *SUC2_prom_::MYB30* on *FT* expression is additive to that of CO, which is obviously much stronger (∼40-fold at ZT16). This suggests that under LDs, although CO is the main activator of *FT*, MYB30 can activate *FT* through a parallel pathway (Figure 5a).

### MYB30 Induces 1.0 kb *FT* Promoter Activity in Transient Bombardment Assay of Leaves

**Figure 6:**
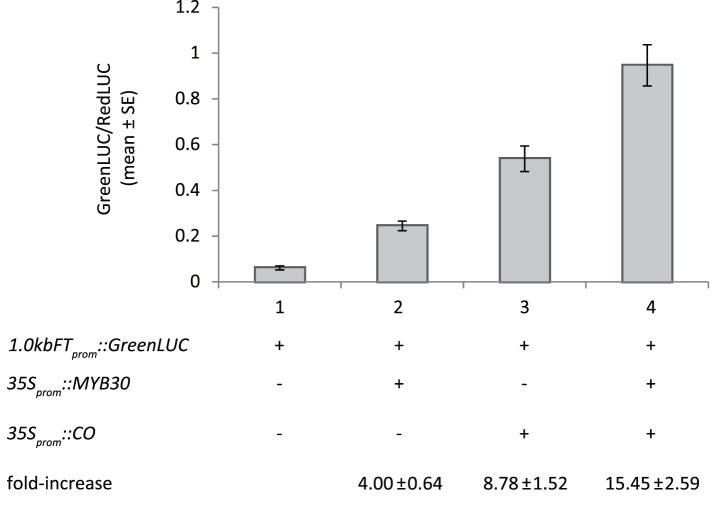
MYB30 increases 1.0 kb *FT* promoter activity. Leaves from Col plants grown in SDs were bombarded with particles carrying *1.0kbFT_prom_::GreenLUC* combined with *35S_prom_::MYB30,35S_prom_::CO* or both as indicated. *35S_prom_::RedLUC* was included to measure transformation efficiencies. Values are shown as relative GreenLUC compared to RedLUC signals (top panel) after a 16 h–24 h incubation in constant light conditions. Fold-induction over the baseline level obtained for *1.0kbFT_prom_::GreenLUC* alone is indicated in the table below the graph. Values are averages of measurements of five independent leaves from two technical replicates of one bombardment experiment. The experiment was repeated three times with similar results.

To test *FT* promoter activity in association with MYB30 and CO, we used transient bombardment of Arabidopsis leaves. In this assay, a 1 kb *FT* promoter was previously shown to be inducible by CO [7]. Co-bombardment of either CO or MYB30 alone with the 1 kb *FT* reporter showed that MYB30 alone could activate the expression of the reporter although the effect of CO was stronger (Figure 6). Combining MYB30 and CO in the bombardment additively increased the fold-expression of the promoter over the control (Figure 6). Thus, the bombardment assays confirmed that MYB30 and CO can affect *FT* expression in parallel pathways.

### MYB30 Promotes Flowering Independently of SA Levels and FLC

**Figure 7:**
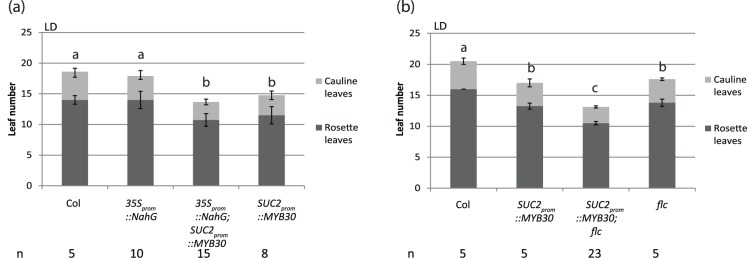
*SUC2_prom_::MYB30* promotes flowering independently of SA and FLC. (**a**) Flowering time measurement of *Col*, *NahG*, *SUC2_prom_::MYB30* and *NahG;SUC2_prom_::MYB30* under LD conditions. (**b**) Flowering time measurement of Col, *SUC2_prom_::MYB30*, *flc* and *SUC2_prom_::MYB30;flc* under LD conditions**.** Statistical significance was determent using one way Analysis of Variance (ANOVA) followed by multiple comparison with the Holm-Sidak procedure (p<0.01). Significant differences are indicated by different letters above the bars. The number of plants for each genotype (n) is indicated below the graph.

MYB30 has previously been shown to be a positive regulator of pathogen defense, which is in part explained through increased accumulation of SA upon increased *MYB30* expression [38]. In addition, MYB30 may directly regulate genes in the SA signaling pathway. SA was shown to be required for accelerated flowering observed in *siz1* mutants [34] and the absence of SA in NahG plants was shown to delay flowering compared to Col-0 plants in LD and SD [32]. To test whether MYB30 promotes flowering through an SA pathway and dependent on FLC, we introduced the *SUC2_prom_::MYB30* construct into *NahG* and *flc3* mutant backgrounds by crossing. In our LD growth conditions, flowering was not changed significantly in the SA-deficient *35S_prom_::NahG* plants as compared to WT. In addition, *SUC2_prom_::MYB30;35S_prom_::NahG* plants flowered as early as *SUC2_prom_::MYB30* plants, which suggested that MYB30 accelerated flowering independently of its reported effect on increasing SA levels (Figure 7a). Expression analysis of *FT*, *TSF* and the pathogenesis response marker gene *PR1* further supported the flowering-time data and confirmed a role of MYB30 in inducing *PR1* expression (Figure S5). *FT* was super-induced at ZT16 in *SUC2_prom_::MYB30;35S_prom_::NahG* plants confirming that SA was not required for the effect of MYB30 on *FT* expression (Figure S5). As before, strong *MYB30* expression in the phloem repressed the expression of *TSF*, a response which was also detected in the *NahG* background (Figure S5).

As indicated by the unaltered expression of *FLC* in *SUC2_prom_::MYB30* expressing plants (Figure 3), early flowering mediated by MYB30 was not dependent on FLC. Compared to the flowering observed in both parents, *SUC2_prom_::MYB30;flc3* double mutants showed an additive early flowering phenotype (Figure 7b).

In conclusion, increased expression of *MYB30* in the phloem can modulate flowering by increasing the expression of *FT* through a pathway that acts either independent of or additively to CO. Increased *FT* expression by MYB30 is independent of *flc* and does not require the presence of SA.

## Discussion

### Working Model for the Effect of MYB30 on Flowering Time

MYB30 can enter the flowering-time regulatory network by influencing the transcription rate of *FT*. As suggested by transient expression assays, the activation of *FT* by MYB30 requires sequences contained in the proximal 1 kb of the *FT* promoter. It is unclear, whether *FT* is a direct target of MYB30 or whether other factors mediate between the transcription factor and *FT*. In transgenic plants, MYB30 activates *FT* in absence of CO approximately 5-fold and a co-activation leads to a 5-fold higher induction of *FT* over CO-induced levels. This additive induction indicates that CO and MYB30 represent parallel inputs for *FT* induction. Although *MYB30* overexpression reportedly leads to increased SA levels, which have been shown to accelerate flowering, we showed that MYB30-mediated induction of *FT* in the phloem does not depend on the presence of SA (Figure 7; [32], [39]). Furthermore, neither *SVP* nor *FLC* transcript levels are significantly altered in *SUC2_prom_::MYB30* plants at different developmental stages and we have shown that the induction of *FT* by MYB30 is independent of *FLC* (Figures 3 and 7).

### Opposed Effects of MYB30 on the Expression of *FT* and *TSF*

Under LD conditions, *FT* and *TSF* are affected by MYB30 overexpression but their transcripts are regulated in opposite patterns in *SUC2_prom_::MYB30* plants (Figure 5). The accelerated flowering observed in wild-type Col and *co* mutant plants expressing *SUC2_prom_::MYB30* regulate is explained through an activation of *FT*, which is epistatic to the repression of *TSF.* In contrast, the delay of flowering visible in *SUC2_prom_::MYB30*;*ft* mutant plants is explained by the repression of *TSF* (Figure 4 and 8).

*FT* and *TSF* are paralogous genes in Arabidopsis and both encode for proteins that contribute to the florigen function [3], [26], [27], [28]. In LDs, genetic defects of *FT* are epistatic to those of *TSF* and this is correlated with the significantly higher expression levels of the former in LDs. Both genes show substantial conservation in their proximal promoter sequences, but *TSF* lacks the distal enhancer that is important for high activation of *FT* by CO [7]. Nevertheless, *FT* and *TSF* are usually co-regulated and share CO as upstream activator as well as SVP and FLC as transcriptional repressors [26], [28]. Based on *promoter::GUS* reporter plants, both genes are predominantly expressed in the phloem but *FT* expression is usually restricted to the distal leaf veins, whereas *TSF* mainly expresses in the veins of hypocotyls and leaf petioles [28].

MYB30 is the first upstream factor that regulates *FT* and *TSF* transcript levels in an obvious opposite way, where *FT* is up-regulated and *TSF* is down-regulated (Figure 3). FT and TSF have redundant and independent roles in the floral transition. For example, TSF but not FT is required for the acceleration of flowering in response to external cytokinin application and only *TSF* expression is increased in these conditions [49].

High *FT* levels may also cause *TSF* repression. Such a negative feed-back has been observed in a previous study where *TSF* was down-regulated in plants that contained an activation-tagged allele of *FT*. This is also supported by our observation that *TSF* levels were increased in the *ft* mutant compared to WT (Figure 5b). In contrast, *FT* expression was not obviously changed when *TSF* was activated [26], [27]. Neither *ft* nor *tsf* loss-of function mutants show altered expression of their respective paralog but this could be due to the small overlap of their expression domains [28].

### MYB30 as a New Component in Flowering Time Control?

Our data show that high levels of the transcription factor MYB30 in the phloem companion cells affect flowering time (Figure 1). Based on GUS signals controlled by the upstream intergenic region of *MYB30*, the gene is present in the phloem in wild-type plants (Figure S3). In addition, the diurnal expression pattern of *MYB30* mRNA levels showed a peak at ZT16 in LDs, which overlaps with the moment of the strongest *FT* induction by CO in LDs (Figure 2; [15], [50]). Since *MYB30* expression is induced by biotic stress [38], it is possible that accelerated flowering observed under stress condition may be dependent on MYB30. Stress accelerated flowering has been reported in Arabidopsis as response to nutrient depletion [51], pathogen perception [52], temperature [53], UVC-stress and external SA application [32]. In *Pharbitis nil* and *Lemna paucicostata*, induction of flowering in response to nutrient depletion was abolished by the addition of aminooxyacetic acid, an effect that was reversed by adding SA externally [54], [55], [56]. However, we could not confirm the delayed flowering that was reported earlier for *35S_prom_::NahG* plants (Figure 7; [32]). In addition, we did not observe a delay in flowering in two independent *myb30* loss-of-function mutants grown under normal LD and SD greenhouse conditions (Figure 1). It is probable that multiple factors act together to cause a distinct stress-induced acceleration of flowering and as long as these factors are not entirely known, different experimental observations are expected considering variant culture conditions between laboratories.

Alternatively, a more constitutive role of *MYB30*-like genes could be masked by redundancy within the large MYB transcription factor family. MYB30 is part of a clade of 10 proteins, with MYB96 and MYB94 as closest relatives [57]. Similarly, regulation of *FT* by *CCAAT*-box binding NF-Y complexes, which are encoded by multi-gene families, was first shown by ectopic expression and required the generation of multiple stacked loss-of-function lines to demonstrate their role as genuine components of the photoperiod pathway [16], [17], [47].

In conclusion, the multi-pathway regulator MYB30 seems well positioned to connect gene networks regulating flowering, hormone signaling and stress perception, because it regulates genes involved in these various pathways and perceives signal inputs from the stress-related pathways. Further work will first have to focus on determining the conditions under which cross-talk between the signals is strongest, which will greatly facilitate uncovering whether MYB30 truly plays a biologically relevant role in flowering.

## Supporting Information

Figure S1 **Two independent transgenic lines of** ***SUC2_prom_::MYB30*** **flower early in LDs.** Two transgenic lines of *SUC2_prom_::MYB30* and *Col-0* were grown in LDs, and their rosette and cauline leaves were counted. Statistical significance was determined using the Student’s t-test (p<0.01). Significant differences are indicated by different letters above the bars. The number of plants for each genotype (n) is indicated below the graph.
(EPS)

Figure S2 **GUS staining of** ***MYB30_prom_::GUS*** **plant.** 10-day-seedlings transformed with *MYB30_prom_::GUS* in LDs were stained in X-GLUC solution to detect GUS activity.
(EPS)

Figure S3 **Diurnal expression of** ***FT*** **and** ***CO*** **–2^nd^ biological replicate. (a)** *FT* expression was measured comparing WT to *SUC2_prom_::MYB30* expressing Col plants. 12-day-plants in LDs were collected every 4 hours from ZT0-ZT24. **(b)** *CO* expression was measured comparing WT to *SUC2_prom_::MYB30* expressing Col plants. 12-day-plants in LDs were collected every 4 hours from ZT0-ZT24. Error bars represent the standard error of three technical replicates relative to the expression *PP2A* (AT1G13320).
(EPS)

Figure S4 **Phenotype of** ***SUC2_prom_::MYB30*** **;** ***ft;tsf*** **plants grown in LDs.**
(EPS)

Figure S5 ***SUC2_prom_::MYB30*** **promotes flowering independently of SA.** *FT* **(a)**, *TSF* **(b)** and *PR1* **(c)** mRNA levels were measured in *Col*, *NahG*, *SUC2_prom_::MYB30* and *NahG;SUC2_prom_::MYB30* plants. Samples were collected at ZT 16 from 10-day old LD grown seedlings.
(EPS)

Table S1 **List of oligonucleotides used in this study.**
(DOCX)
