# Supplementary material for: Elevated Levels of MYB30 in the Phloem Accelerate Flowering in Arabidopsis through the Regulation of FLOWERING LOCUS T
Source: PLoS One. 2014 Feb 25;9(2):e89799. doi: 10.1371/journal.pone.0089799 (PMC3934951; doi:10.1371/journal.pone.0089799)
Supplement: Table S1 — List of oligonucleotides used in this study. (DOCX) [file pone.0089799.s006.docx]

| **Purpose** | **Primer name** | **Sequence (5’ to 3’)** |
| --- | --- | --- |
| **Expression analysis** | PP2A-RT-fw | AAATACGCCCAACGAACAAA |
|  | PP2A-RT-re | CAGCAACGAATTGTGTTTGG |
|  | FT_cDNA_RT_fw | GGTGGAGAAGACCTCAGGAA |
|  | FT_cDNA_RT_re | ACCCTGGTGCATACACTGTT |
|  | MYB30_RT_fw | ACTGGTGATTTGCGGACT |
|  | MYB30_RT_re | CAAGAGTGATGATGGGAAGG |
|  | TSF_RT_fw | CACGAGGTTGGTCTCTCTTAAG |
|  | TSF_RT_re | CTGGCAGTTGAAGTAAGAG |
|  | CO-RT-fw | TAAGGATGCCAAGGAGGTTG |
|  | CO-RT-re | CCCTGAGGAGCCATATTTGA |
|  | SVP-RT-fw | GAAGGACAGTCGTCGGAGTC |
|  | SVP-RT-re | GCCTCTTCCATAGGCAGAAA |
|  | FLC-RT-fw | ACAAAAGTAGCCGACAAGTCACCT |
|  | FLC-RT-re | GGAAGATTGTCGGAGATTTGTCCA |
|  | MYB30_PCR_fw | GGTGAGGCCTCCTTGTTGT |
|  | MYB30_PCR_re | TGAGTCTTCGGCGAGTTTTT |
|  | PR1-RT-fw | TTCTTCCCTCGAAAGCTCAA |
|  | PR1-RT-re | AAGGCCCACCAGAGTGTATG |
|  | oligo_dT18 | TTTTTTTTTTTTTTTTTT |
| **Genotyping** | myb30-2_GK022F04_fw | ACCTTTGTCGCCAAGCTTAAC |
|  | myb30-2_GK022F04_re | ACCCGCTAGCTGAGGAAGTAG |
|  | GABI | ATATTGACCATCATACTCATTGC |
|  | NahG_fw | ACTCTGCCGCTACTCCCATA |
|  | NahG_re | GAGATGAAAGCCACCACGTT |
|  | myb30-1_SALK_027644C_fw | TCCTTGTTGTGACAAAGGAGG |
|  | myb30-1_SALK_027644C_re | ATGATCAGGTGAAACACCAGC |
|  | ft10_GABI_rp | CAGGTTCAAAACAAGCCAAGA |
|  | ft10_GABI_lp | TAAGCTCAATGATATTCCCGTACA |
|  | ft10_GABI_LB | CCCATTTGACGTGAATGTAGACAC |
|  | tsf1-rp | CTGGCAGTTGAAGTAAGAG |
|  | tsf1-lp | CACGAGGTTGGTCTCTCTTAAG |
|  | LBb1.3 | ATTTTGCCGATTTCGGAAC |
|  | co-sail-fw | AGATTCTGCCTACTTGTGCATG |
|  | co-sail-re | CTCTTCTCTGGATCGGTCATTG |
|  | co-sail_Garlic_LB3 | TAGCATCTGAATTTCATAACCAATCTCGATACAC |
| **Plasmid construction** | MYB30prom_fw | GGGGACAAGTTTGTACAAAAAAGCAGGCTTCGTTACAGTCCTCCTATCT |
|  | MYB30prom_re | GGGGACCACTTTGTACAAGAAAGCTGGGTCTATGATCTTGAACTCCCT |
|  | 1kbFTp-GW-fw | GGGGACAAGTTTGTACAAAAAAGCAGGCTATAATATGGCCGCTTGTTTATA |
|  | 1kbFTp-GW-re | GGGGACCACTTTGTACAAGAAAGCTGGGTCTTTGATCTTGAACAAACAGGT |
